# Early Developmental Marginal Zinc Deficiency Affects Neurogenesis Decreasing Neuronal Number and Altering Neuronal Specification in the Adult Rat Brain

**DOI:** 10.3389/fncel.2019.00062

**Published:** 2019-03-01

**Authors:** Ana M. Adamo, Xiuzhen Liu, Patricia Mathieu, Johnathan R. Nuttall, Suangsuda Supasai, Patricia I. Oteiza

**Affiliations:** ^1^Department of Biological Chemistry and IQUIFIB (UBA-CONICET), Facultad de Farmacia y Bioquimica, Universidad de Buenos Aires, Buenos Aires, Argentina; ^2^Department of Nutrition and Department of Environmental Toxicology, University of California, Davis, Davis, CA, United States; ^3^Department of Molecular Tropical Medicine and Genetics, Faculty of Tropical Medicine, Mahidol University, Bangkok, Thailand

**Keywords:** zinc, brain development, ERK1/2, Tbr2, zinc deficiency

## Abstract

During pregnancy, a decreased availability of zinc to the fetus can disrupt the development of the central nervous system leading to defects ranging from severe malformations to subtle neurological and cognitive effects. We previously found that marginal zinc deficiency down-regulates the extracellular signal-regulated kinase 1/2 (ERK1/2) signaling pathway and affects neural progenitor cell (NPC) proliferation. This study investigated if marginal zinc deficiency during gestation in rats could disrupt fetal neurogenesis and affect the number and specification of neurons in the adult offspring brain cortex. Rats were fed a marginal zinc deficient or adequate diet throughout gestation and until postnatal day (P) 2, and subsequently the zinc adequate diet until P56. Neurogenesis was evaluated in the offspring at embryonic day (E)14, E19, P2, and P56 measuring parameters of NPC proliferation and differentiation by Western blot and/or immunofluorescence. At E14 and E19, major signals (i.e., ERK1/2, Sox2, and Pax6) that stimulate NPC proliferation and self-renewal were markedly downregulated in the marginal zinc deficient fetal brain. These alterations were associated to a lower number of Ki67 positive cells in the ventricular (VZs) and subventricular zones (SVZs). Following the progression of NPCs into intermediate progenitor cells (IPCs) and into neurons, Pax6, Tbr2 and Tbr1 were affected in the corresponding areas of the brain at E19 and P2. The above signaling alterations led to a lower density of neurons and a selective decrease of glutamatergic neurons in the young adult brain cortex exposed to maternal marginal zinc deficiency from E14 to P2. Current results supports the concept that marginal zinc deficiency during fetal development can disrupt neurogenesis and alter cortical structure potentially leading to irreversible neurobehavioral impairments later in life.

## Introduction

Prenatal zinc deficiency resulting from insufficient dietary intake, absorption, or transport can compromise development of the central nervous system leading to a spectrum of defects ranging from severe congenital malformations to subtle neurological and cognitive impairments. Severe zinc deficiency during fetal development has been implicated as a mechanism contributing to neural tube defects (NTDs). Severe dietary zinc deficiency in rats during pregnancy leads to NTDs in association with decreased cell proliferation in the ventricular zone (VZ) of the fetal brain (Swenerton et al., 1969). In humans, supplementation with dietary zinc and adequate plasma zinc concentrations are related to a reduced risk of NTDs (Velie et al., 1999; Dey et al., 2010). Although developmental marginal zinc deficiency does not cause gross malformations like NTDs, it is associated with neurological morbidity such as impairments in learning, working memory, and social behavior (Hagmeyer et al., 2015). Similar cognitive defects result from secondary zinc deficiency caused by gestational exposure to infection in rats (Kirsten et al., 2015).

Developmental exposure to a decreased zinc availability could have a long-term and irreversible impact on the offspring’s brain leading to neurological and behavioral disorders later in life. In rats, severe postnatal zinc deficiency impairs neurogenesis in the cerebellum (Dvergsten et al., 1983), and decreases the expression of genes related to proliferation and neuronal differentiation in the hippocampus (Gower-Winter et al., 2013). In cultured cells, zinc deficiency impairs human IMR-32 neuroblastoma cell proliferation and induces apoptosis (Adamo et al., 2010), and inhibits retinoic acid-induced neuronal differentiation (Gower-Winter et al., 2013). Furthermore, zinc deficiency alters brain redox regulation and affects signaling pathways involved in neurogenesis (Zago et al., 2005; Aimo et al., 2010a,b; Mackenzie et al., 2011). Thus, we previously observed that gestational marginal zinc deficiency in rats decreases the number of neuronal progenitor cells (NPCs) expressing Ki67 in the VZ at embryonic day (E) 19 (Nuttall et al., 2015). NPCs give rise to all neuronal types present in the brain and play a pivotal role in regulating the balance between cell self-renewal and neurogenesis. Cortical excitatory neurons are directly generated from radial glial progenitors (RGPs) during embryonic neurogenesis or indirectly through intermediate progenitor cells (IPCs) derived from the VZ (Haubensak et al., 2004; Noctor et al., 2004). Radial glia proliferate at the ventricular surface and IPCs divide at a non-surface area in the VZ and subventricular zone (SVZ). The transcription factor cascade involving Pax6, Tbr2 and Tbr1 regulates the different stages of differentiation from NPCs to IPCs to cortical neurons. Tbr1 and Tbr2 are part of the T-box transcription factor subfamily Tbr1, and play key roles in glutamatergic neuron differentiation in the cerebral cortex, olfactory bulbs, hippocampal dentate gyrus, cerebellum and adult SVZ (Mihalas and Hevner, 2017). The extracellular signal-regulated kinase (ERK1/2) pathway is also a key regulator of NPC proliferation and neuronal differentiation (Nuttall and Oteiza, 2012). Marginal zinc deficiency during pregnancy in rats causes a decreased phosphorylation of ERK1/2 in the frontal cortex of the fetal rat brain at E19 (Nuttall et al., 2015) which is associated with a decrease in NPC number. However, it remains unclear if this decrease in NPCs can affect the number of neurons in the adult offspring brain as well as the process of neuronal differentiation and specification.

A complex and tightly regulated cascade of events lead to the differentiation of NPCs into neurons. Our previous results showed impaired NPC proliferation in association with maternal zinc deficiency. Thus, the goal of the present work was to evaluate whether a marginal zinc diet fed throughout gestation and until postnatal day (P) 2 can: (i) affect, in the offspring brain, the transcription factor cascade involved in NPC and IPC proliferation and differentiation into glutamatergic neurons; and (ii) cause long-term effects in the number and specification of cortical neurons in the offspring young adult brain. To such end, the temporal activation/expression of ERK1/2 and transcription factors Sox2, Pax6, Tbr2 and Tbr1 were characterized in the E14, E19 and P2 offspring brain. Alterations in cortical neuronal number and specification to glutamatergic and GABAergic neurons secondary to maternal marginal zinc deficiency were assessed in the adult offspring brain after postnatal repletion of dietary zinc.

## Materials and Methods

### Materials

Primary antibodies for β-actin (#12620), phospho (Thr202/Tyr204) ERK (#4370), and ERK (#9102), were from Cell Signaling Technology (Danvers, MA, USA). Antibodies for glutamic acid decarboxylase 65 (GAD65; SC-377154) and Tbr1 (SC-376258; Western blot) were from Santa Cruz Biotechnology (Santa Cruz, CA, USA). Antibodies for Neu-N (MAB377) and Tbr2 (AB15894) were from Millipore (Burlington, MA, USA). The antibody for Ki67 (550609) was obtained from BD Pharmingen (San José, CA, USA). Antibodies for Sox2 (ab97959), Tbr1 (ab31941; immunofluorescence), vGlut1 (ab77822) and Pax6 (ab5790) were from Abcam Inc. (Cambridge, MA, USA). Secondary fluorescent antibodies were obtained from Jackson ImmunoResearch Co. Laboratories (West Grove, PA, USA). Polyvinylidene difluoride (PVDF), membranes and molecular weight standards for Western blot were obtained from BIO-RAD (Hercules, CA, USA). The enhanced chemiluminescence (ECL) Western blotting system was from Thermo Fisher Scientific Inc. (Piscataway, NJ, USA). Zinquin, the antibody for γ amino butyric acid (GABA; A2052), and all other reagents were of the highest quality available and were purchased from Sigma-Aldrich Co. (St. Louis, MO, USA).

### Animals and Animal Care

**Figure 1:**
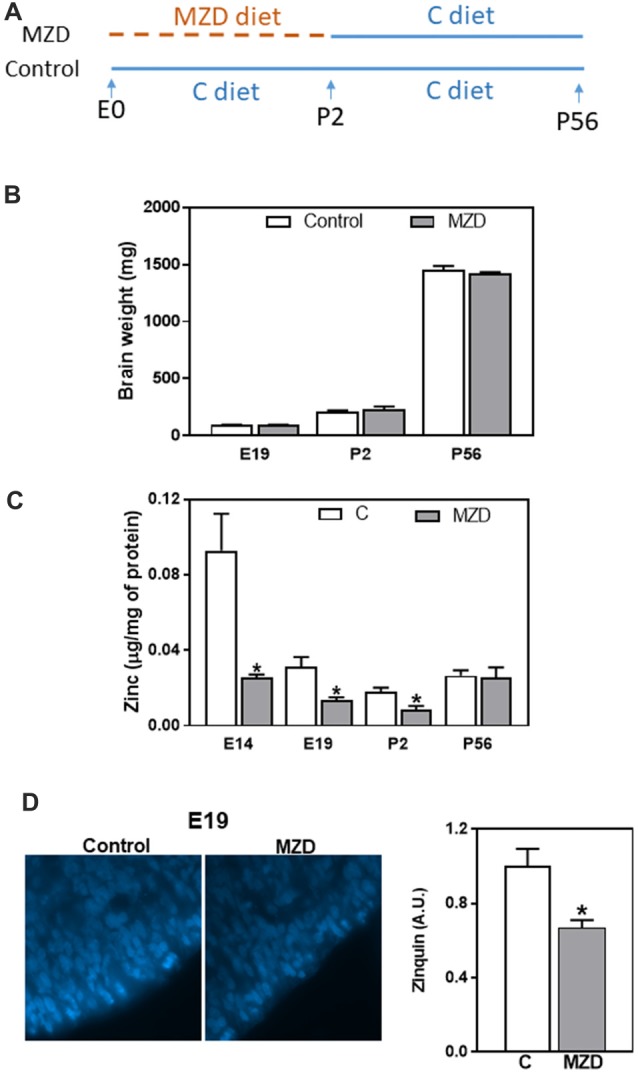
Fetal/offspring brain weight and zinc concentration after maternal consumption of a control or a marginal zinc diet throughout gestation and until postnatal day (P) 2. **(A)** Experimental design. **(B)** Embryonic day (E) 19–P56 offspring brain weight. **(C)** Zinc concentration in fetal/offspring cortical tissue (CT) and brain cortex 100,000× *g* supernatants were measured by atomic emission spectroscopy (AES). **(D)** Labile zinc was measured in the ventricular zone (VZ) at E19 by zinquin staining (blue fluorescence). Micrographs show the VZ of the dorsomedial frontal cortex at 1,000-fold magnification. Fluorescence was quantified as described in “Materials and Methods” section. **(B–D)** Data are shown as mean ± SEM and are the average of 4–6 litters per group. *Significantly different from the control group at the same developmental stage (*t*-test, *p* < 0.05).

All procedures were in agreement with standards for the care of laboratory animals as outlined in the National Institutes of Health Guide for the Care and Use of Laboratory Animals. All procedures were administered under the auspices of the Animal Resource Services of the University of California at Davis, which is accredited by the American Association for the Accreditation of Laboratory Animal Care. Experimental protocols were approved before implementation by the University of California at Davis Animal Use and Care Administrative Advisory Committee and were administered through the Office of the Campus Veterinarian. Adult Sprague-Dawley rats (Charles River, Wilmington, MA, USA; 200–225 g) were housed individually in stainless steel cages in a temperature—(22–23°C) and photoperiod—(12-h light/dark) controlled room. An egg-white protein-based diet with adequate zinc (25 μg zinc/g) was the standard control diet (Keen et al., 1989). Animals were fed the control diet for 1 week before breeding. The overall experimental design is shown in Figure 1A. Males and females were caged together overnight and the following morning (E0), after the presence of a sperm plug confirmed a successful breeding, female rats (six animals/group for E14, E19, and P2/P56) were divided into two groups and fed *ad libitum* a control diet (25 μg zinc/g diet, control group) or a diet containing a marginal concentration of zinc (10 μg zinc/g diet; MZD group). Food intake was recorded daily, and body weight was measured at 3-day intervals. On E14 and E19, dams were anesthetized with isoflurane (2 mg/kg body weight) and laparotomies were performed. The gravid uterus was removed and fetuses were weighed. Fetal brains were removed, weighed and immediately either processed for immunohistochemistry, or removed and kept on ice to microdissect regions enriched in cortical tissue (CT), which were then frozen in liquid nitrogen and stored at −80°C. In the E14 offspring, CT included the cortical neuroepithelium with the SVZ and VZ; in E19 offspring, CT included the cortical plate with the SVZ and VZ. At P2, litters were adjusted to eight pups/litter, and brain/brain cortices were dissected from the euthanized pups and processed as described before. Dams from both the control and the MZD groups were subsequently fed the control diet. Pups were weaned at P21 and were all fed the control diet until P56 when euthanized; blood and brain/brain cortex collection was carried out as described before.

With regard to the time of exposure of the offspring to zinc deficiency, it should be considered that milk zinc content does not decrease in conditions of marginal zinc nutrition both in rodents and humans, even when maternal plasma zinc levels are low (Kelleher and Lönnerdal, 2005). Thus, in the current experimental model, it is expected that the offspring would have access to similar amounts of zinc in milk starting at birth in both control and MZD groups.

### Determination of Zinc Concentrations

The concentration of zinc in diets and brain supernatants was measured by inductively coupled plasma atomic emission spectroscopy (ICP-AES) as described by Clegg et al. (2005). E14 and E19 brain CT and P2 and P56 cortices were weighed, homogenized in ice-cold PBS (1:10), and centrifuged for 60 min at 100,000× *g* at 4°C. The supernatant was collected and protein concentration was measured using the Bradford assay (Bradford, 1976). Three milliliter of 16 N HNO_3_ were added to the 100,000× *g* supernatants and diet samples and allowed to digest for 72 h. Samples were dried, and resuspended in ultrapure water. Zinc concentration was determined by inductively coupled plasma atomic emission spectroscopy (ICP-AES; Trace Scan; Thermo Elemental, Franklin, MA, USA). Certified reference solutions (QC 21; Spec CentriPrep, Metuchen, NJ, USA) were used to generate standard curves. A sample of a National Bureau of Standards bovine liver (SRM1577; U.S. Department of Commerce, National Bureau of Standards, Washington, DC, USA) was included with the samples to ensure accuracy and reproducibility.

### Zinquin Staining

Labile zinc was measured in coronal sections from the fetal rat brain at E19. Slides were overlaid with a solution of 25 μM zinquin in Hank’s balanced salt solution (HBSS) and incubated at 37°C for 40 min. After washing in HBSS, coverslips were mounted with a solution of 90% glycerol and 10% HBSS and imaged on an Olympus BX50 epifluorescence microscope provided with a Cool-Snap digital camera. Image pro software (Rockville, MD, USA) was used to analyze the resulting micrographs. Three randomly selected fields were measured per animal and experimental condition (*n* = 3).

### Western Blot Analysis

Extraction of total cellular protein from CT and cortex homogenates was done as previously described (Aimo et al., 2010b). Protein concentration was measured using the Bradford assay (Bradford, 1976), and aliquots containing 25–50 μg of protein were separated by reducing 10% (w/v) SDS-PAGE and electroblotted to PVDF membranes. Colored molecular weight standards were run simultaneously. Membranes were blocked for 1 h in 5% (w/v) nonfat milk and incubated overnight with the corresponding primary antibodies (1:1,000–1:5,000) in 1% (w/v) bovine serum albumin in TBST (20 mM Tris, 150 mM NaCl, pH 7.4, 0.1% Tween 20) at 4°C. After incubation for 1 h at room temperature with the corresponding peroxidase-conjugated secondary antibodies (1:10,000–1:30,000), proteins were visualized by chemiluminescence detection, and subsequently quantified, using a Phosphorimager 840 (Amersham, Piscataway, NJ, USA).

### Immunofluorescence

E14, E19, and P2 rat brains were dissected out and fixed in a 4% (w/v) solution of paraformaldehyde in PBS overnight. P56 rats were deeply anesthetized as described above and perfused transcardially with PBS followed by 4% (w/v) solution of paraformaldehyde in PBS, after which brains were collected. In all cases, tissue cryoprotection was subsequently performed in 30% (w/v) sucrose until the tissue sank down, after which, brains were submerged in Cryoplast freezing medium (Biopack, Buenos Aires, Argentina), frozen, cut into 18 μm coronal sections on a Leica CM 1850 cryotome (Leica Microsystems, Nussloch, Germany), and mounted on positively charged microscope slides. Sections were blocked for 45 min in 1% (v/v) donkey serum in 0.1% (v/v) Triton X-100 in PBS, and incubated with the corresponding dilution of primary antibody in blocking solution (1:200 rabbit anti-Sox2, 1:100 rabbit anti-Pax6, 1:200 rabbit anti-Trb1, 1:100 chicken anti-Tbr2, 1:200 mouse anti-NeuN, 1:200 rabbit anti-vGlut1, 1:200 rabbit anti-GABA, 1:100 mouse anti-Ki67) overnight at 4°C. Sections were then washed once in 0.1% (v/v) Triton X-100 in PBS and once in 0.1 M phosphate buffer, pH 7.4, and incubated with the corresponding dilution of secondary antibody (1:500 Cy3-conjugated donkey anti-rabbit, 1:500 Alexa 488 donkey anti-mouse IgG, and 1:500 Alexa 488 donkey anti-chicken) for 2 h at room temperature. After immunostaining, cell nuclei were stained with 1 μg/ml Hoechst 33342 and sections were imaged using an Olympus FV 1000 laser scanning confocal microscope or an Olympus BX50 epifluorescence microscope provided with a Cool-Snap digital camera. Image pro software (Rockville, MD, USA) was used to merge and analyze the resulting micrographs. Marker-positive cells were counted using ImageJ (National Institutes of Health, Bethesda, MD, USA) and results were expressed as the number of positive cells per area. At least 300 cells were counted for each cell marker, analyzed in three independent experiments that were performed in triplicates for four animals per group. Alternatively, fluorescence intensity was measured for vGlut1 and GABA.

### Statistical Analysis

Data for the control and MZD groups at each developmental stage were analyzed by Student’s *t*-test using Statview 5.0 (SAS Institute Inc., Cary, NC, USA). The litter was the statistical unit. A *p* value < 0.05 was considered statistically significant. Data are shown as mean ± SE.

## Results

### Animal Outcome

Pregnant dams were fed a marginal zinc or a control diet from E0 until P2. Subsequently all dams until P21, and the offspring, until P56, were fed the control diet (Figure 1A). As previously described (Aimo et al., 2010b; Nuttall et al., 2015), we observed that a marginal zinc nutrition throughout gestation does not affect overall maternal and fetal outcome (data not shown). Furthermore, consumption of the marginal zinc diet throughout gestational and perinatal period did not affect fetal/offspring brain weight between E19 and P56 (Figure 1B). Zinc concentration in E14 and E19 CT and P2 brain cortex 100,000× *g* supernatants was significantly lower (72%, 58% and 56%, respectively) in MZD offspring compared to the control group (Figure 1C). Loosely bound zinc in the E19 brain was evaluated by Zinquin staining and subsequent fluorescence microscopy. In the control group, Zinquin fluorescence appeared strongest in the VZ and surrounding blood vessels. In the MZD offspring VZ, the intensity of Zinquin fluorescence was 34% lower than in controls (Figure 1D).

### Maternal Marginal Zinc Deficiency Affects Markers of Neurogenesis at E14

**Figure 2:**
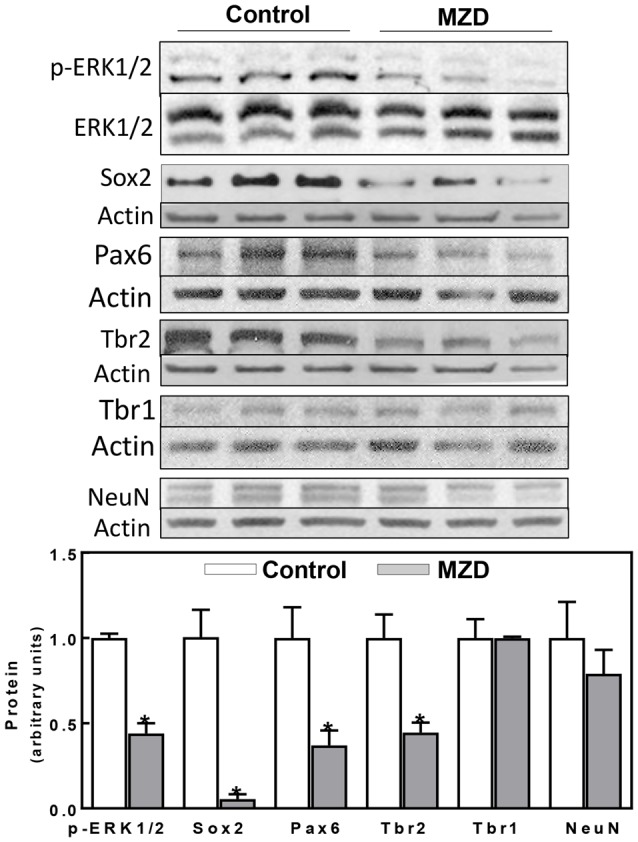
Maternal marginal zinc deficiency affects parameters of neurogenesis in the E14 rat brain. Phospho extracellular signal-regulated kinase 1/2 (ERK1/2), ERK1/2, Sox2, Pax6, Tbr2, Tbr1 and NeuN protein levels were measured by Western blot in E14 brain CT homogenates. After quantification of bands, phospho-ERK1/2 levels were referred to total ERK1/2 content and the other proteins were referred to β-actin levels. Values (arbitrary units, AUs) were normalized to those of the control group (1) Results are shown as mean ± SE of E14 brain cortices from six litters/group. *Significantly different from the control group (*p* < 0.05, Student’s *t*-test).

Given our previous work showing a decrease in ERK1/2 activation together with a reduction in NPC proliferation, we aimed to investigate the impact of these events on cortical excitatory neurons. Signaling cascades involved in NPC proliferation, self-renewal, and progression to differentiation were evaluated at E14 (Figure 2). ERK1/2 phosphorylation was 66%, lower in the MZD E14 brain CT compared to controls (Figure 2). Maternal marginal zinc nutrition also affected different markers of NPC proliferation and progression to differentiation as evaluated by Western blot (Figure 2). Sox2 is a transcription factor that controls the self-renewal of NPCs throughout development. Maternal marginal zinc deficiency caused a 95% decrease in E14 cortical Sox2 levels compared to controls. Pax6, a transcription factor that is central to the development of the brain cortex, showed 63% lower levels in the E14 cortex from MZD compared to the control group (Figure 2). Marginal zinc nutrition also affected the abundance of Tbr2, a transcription factor that regulates the specification of the intermediate neural progenitors (INPs) that will differentiate into excitatory neurons. Tbr2 levels were 55% lower in MZD compared to control E14 CT. Protein levels of Tbr1 and of the marker of post-mitotic mature neurons NeuN were similar between groups.

### Maternal Marginal Zinc Deficiency Affects Markers of Neurogenesis in the Offspring Brain at E19 and P2

**Figure 3:**
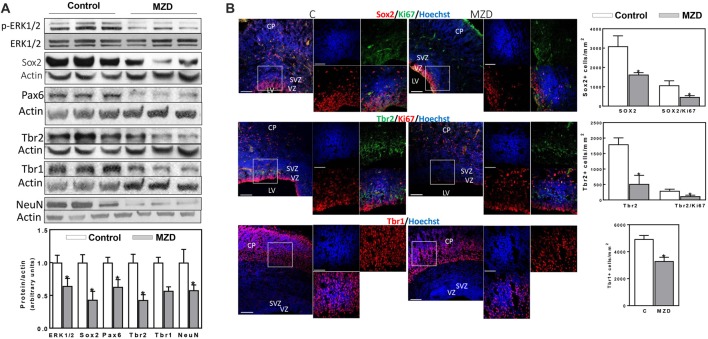
Maternal marginal zinc deficiency affects parameters of neurogenesis in the E19 rat brain. **(A)** Phospho ERK1/2, ERK1/2, Sox2, Pax6, Tbr2, Tbr1 and NeuN protein levels were measured by Western blot in E19 CT homogenates. After quantification of bands, phospho-ERK1/2 levels were referred to total ERK1/2 content and the other proteins were referred to β-actin levels. Values (AUs) were normalized to those of the control group (1) Results are shown as mean ± SE of fetal brain cortices from six litters/group. *Significantly different from the control group (*p* < 0.05, Student’s *t*-test). **(B)** Immunofluorescence for Sox2 (red fluorescence) and Ki67 (green fluorescence); Tbr2 (green fluorescence) and Ki67 (red fluorescence); Tbr1 (red fluorescence) and Ki67 (green fluorescence). Nuclei were visualized with Hoechst staining (blue fluorescence; Scale bar, 100 μm). Images in boxes are shown at a higher magnification (Scale bar, 50 μm). Quantifications were done as described in methods. Results are shown as mean ± SE of E19 brains from four litters/group. *Significantly different from the control group (*p* < 0.05, Student’s *t*-test).

At E19, all measured markers of cortical neurogenesis, from NPC proliferation to fully differentiated neurons, were affected in the MZD embryo brain (Figure 3). ERK1/2 phosphorylation was lower (36%) in the E19 CT from MZD compared to controls. Sox2, Pax6, Tbr2, Tbr1 and NeuN protein levels were 57, 37, 57, 43 and 42% lower in MZD compared to control embryo CT as measured by Western blot (Figure 3A). Immunofluorescence analysis also showed lower levels of total proliferative cells in the E19 SVZ of MZD compared to control offspring (Figure 3B). Thus, NPC Sox2 and IPC Tbr2 positive cells in the SVZ of MZD embryos were 47 and 71%, respectively, compared to controls (Figure 3B). The ratio Sox2/Ki67 and Tbr2/Ki67 positive cells were 56 and 59% lower, respectively, in MZD than in control embryo SVZ. The number of Tbr1 positive cells in E19 cortices were 33% lower in MZD compared to controls.

**Figure 4:**
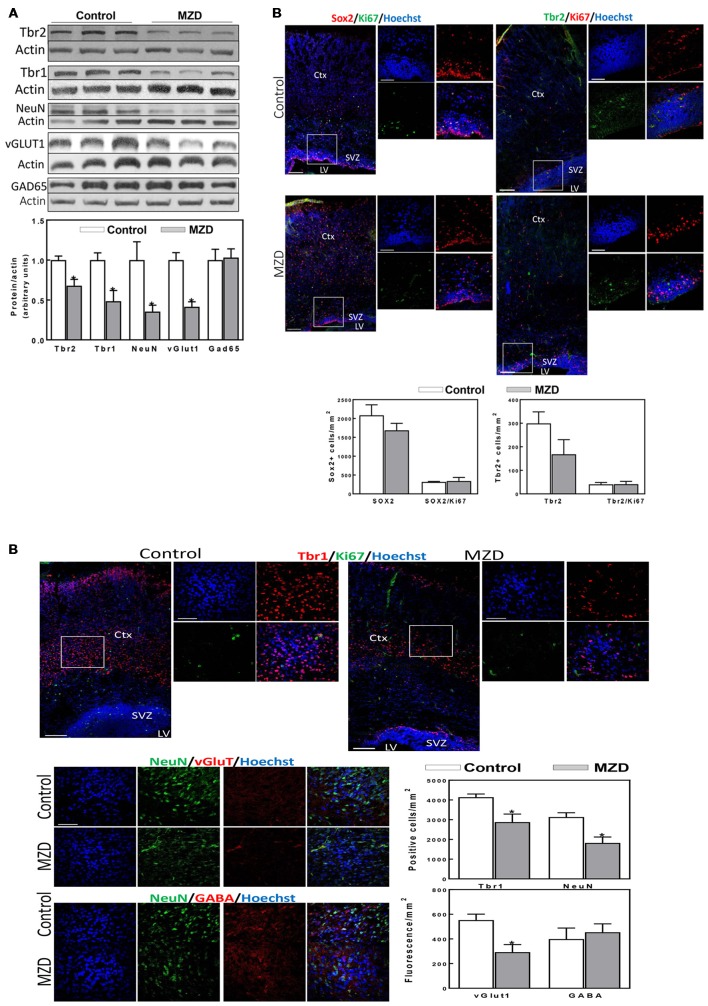
Maternal marginal zinc deficiency affects parameters of neurogenesis in the P2 fetal rat brain. **(A)** Tbr2, Tbr1, NeuN, vGlut1 and glutamic acid decarboxylase 65 (GAD65) protein levels were measured by Western blot in P2 brain cortex homogenates. After quantification of bands, values were referred to β-actin levels and values (AUs) were normalized to those of the control group (1) Results are shown as mean ± SE of fetal brain cortices from six litters/group. *Significantly different from the control group (*p* < 0.05, Student’s *t*-test). **(B)** Immunofluorescence for Sox2 (red fluorescence) and Ki67 (green fluorescence); Tbr2 (green fluorescence) and Ki67 (red fluorescence); Tbr1 (red fluorescence) and Ki67 (green fluorescence); NeuN (green fluorescence) and vGlut1 (red fluorescence); and NeuN (green fluorescence) and γ amino butyric acid (GABA; red fluorescence). Nuclei were visualized with Hoechst staining (blue fluorescence; Scale bar, 100 μm). Images in boxes are shown at a higher magnification (Scale bar, 50 μm). Quantifications were done as described in methods. Results are shown as mean ± SE of offspring brains from four litters/group. *Significantly different from the control group (*p* < 0.05, Student’s *t*-test).

Markers of cortical neurogenesis were measured next at P2 by Western blot (Figure 4A). Tbr2 and Tbr1 levels were significantly lower in MZD than in control brain cortices (32 and 51%, respectively). A decrease in mature neurons is supported by a 64% decrease in NeuN levels in MZD compared to controls. Furthermore, alterations in neuronal specification were already evident at P2. Thus, a 58% decrease in vGlut1, a glutamate/proton exchanger indicator of glutamatergic neuron abundance, was observed in MZD offspring brain cortices. On the other hand, the protein abundance of Gad65 an enzyme involved in GABA synthesis, was similar between groups. Immunofluorescence analysis (Figure 4B) showed no significant differences between groups for Sox2 and Tbr2. Although values were 44% lower for Tbr2, differences were not significant (*p* < 0.15). The number of Tbr1 positive cells was 30% lower in MZD cortices than in controls. The number of NeuN positive cells and of vGlut1 fluorescence intensity were 42 and 47% lower in MZD compared to control P2 offspring brains, while GABA fluorescence intensity was similar between groups.

### Early Developmental Marginal Zinc Deficiency Disrupted Neurogenesis Leading to a Decreased Neuronal Number and Altered Neuronal Specification in the Adult Brain (P56)

**Figure 5:**
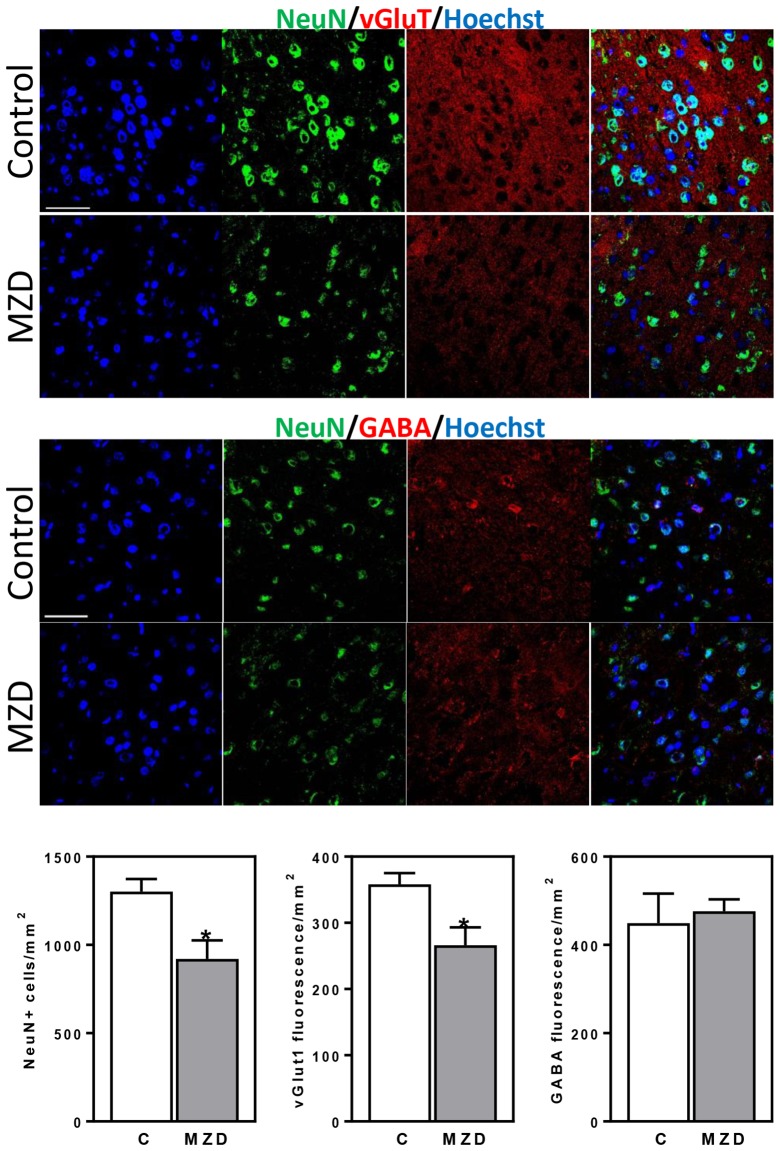
Maternal marginal zinc deficiency from E0 to P2 affects the number and specification of neurons in the young adult brain. NeuN (green fluorescence), vGlut1 (red fluorescence) and GABA (red fluorescence) were measured by immunofluorescence in the frontal cortex at P56. Nuclei were visualized with Hoechst staining (blue fluorescence; Scale bar, 100 μm). Quantifications were done as described in methods. Results are shown as mean ± SE of offspring brain from four litters/group. *Significantly different from the control group (*p* < 0.05, Student’s *t*-test).

We next investigated if the effects of gestational marginal zinc deficiency on fetal brain development have long lasting repercussions, even following repletion with a diet containing adequate zinc. The total number of mature neurons (NeuN positive cells) in the brain cortex of the P56 offspring was evaluated by immunohistochemistry. Marginal zinc deficiency from E0-P2 resulted in a 29% decrease in the number of cells expressing NeuN in the offspring frontal cortex (Figure 5). While no differences in the fluorescence intensity for GABA was observed between groups, 26% lower levels of fluorescence for vGlut1 were observed in the P56 brain cortex from MZD offspring compared to controls (Figure 5).

## Discussion

Results from this study demonstrate that a decreased proliferation of NPCs resulting from gestational marginal zinc deficiency leads to the disruption of cortical neurogenesis in the rat offspring brain. This is associated with a decreased number of neurons in the MZD young adult brain cortex, and an altered specification that results in a reduced number of excitatory glutamatergic neurons, not affecting GABAergic neurons.

Severe nutritional zinc deficiency, both due to low zinc content or high content of zinc-binding phytates, during early development causes NTDs and brain/organ teratology (Oteiza et al., 1990; Velie et al., 1999). Also maternal infections, diabetes, and toxicant exposures that stimulate an acute-phase response causes a decreased transport of zinc to the developing fetus (Nuttall et al., 2017), which can lead to increased risk of NTDs (Chua et al., 2006; Uriu-Adams and Keen, 2010). On the other hand, a marginally low dietary zinc intake during pregnancy is associated with decreased fetal heart rate variability, suggesting impaired regulation of the autonomic nervous system (Spann et al., 2015). Accordingly, prenatal zinc supplementation improves the regulation of the autonomic nervous system later in life (Caulfield et al., 2011). Mild developmental zinc deficiency, both in humans and rodents, impairs learning, working memory, and social behavior (Hagmeyer et al., 2015). Similar cognitive defects result from secondary zinc deficiency in a rat model of maternal infection (Kirsten et al., 2015). Importantly, these cognitive defects are consistent with rodent models of autism spectrum disorder, suggesting that disruption of fetal brain development resulting from marginal zinc deficiency could contribute to the risk of developing autism (Grabrucker et al., 2014; Kirsten et al., 2015; Nuttall, 2015). Moreover, reports in humans have established an association between zinc deficiency and Phelan McDermid Syndrome, a genetic disorder characterized by features of autism spectrum disorders (Pfaender et al., 2017). Thus, although not teratogenic, marginal zinc deficiency can have long-lasting effects on the nervous system.

We currently observed that consumption of a marginal zinc deficient diet during pregnancy did not affect the overall pregnancy and fetal/offspring outcome. On the other hand, the concentration of zinc in the offspring brain cytosolic fraction was markedly affected from E14 through P2. This finding stresses the major impact that a mild decrease in zinc availability can have on zinc homeostasis in the developing brain. Cytosolic zinc and Zinquin-reactive zinc largely reflect loosely bound and rapidly available zinc pools, which are highly relevant to the regulation of cell signaling. For example, and stressing the relevance of available zinc pools, both the decrease in ERK1/2 phosphorylation and cell proliferation observed in zinc-deficient IMR-32 cells are rapidly restored upon zinc supplementation and *via* the inhibition of the ERK1/2-directed phosphatase PP2A (Nuttall et al., 2015).

Zinc deficiency affects signaling pathways that can contribute to altered brain development. In this regard, we previously observed a downregulation of transcription factors NF-κB and NFAT, the activation of MAPKs p38 and JNK, and a downregulation of ERK1/2 both in zinc deficient IMR-32 cells and E19 fetal rat brain (Zago et al., 2005; Aimo et al., 2010b; Nuttall et al., 2015). In particular, ERK1/2 phosphorylation showed a major decrease in the MZD brain CT at E14 and E19. This can in part explain maternal zinc deficiency-associated decrease in VZ NPC number and proliferation and the observed alterations in neuronal specification. In this regard, ERK1/2 is not only important for NPC proliferation but also for neuronal differentiation (Samuels et al., 2008; Pucilowska et al., 2012). In humans, mutations that disrupt ERK1/2 signaling impair brain development leading to cognitive dysfunction in neuro-cardio-faciocutaneous syndromes and autism spectrum disorders (Samuels et al., 2009; Kalkman, 2012). Mice with a conditional deletion of ERK1/2 in NPCs have decreased NPC proliferation leading to abnormal distribution of neurons in the cortical plate, increased excitability of cortical neurons, increased anxiety-like behavior, reduced memory, and impairments of social behavior (Satoh et al., 2011; Pucilowska et al., 2012). Overall, ERK1/2 can be a key signal underlying the altered neurogenesis and behavior associated with maternal marginal zinc deficiency (Nuttall and Oteiza, 2012).

Transcription factor Sox2 functions by regulating NPC self-renewal in both the developing and mature brain, and inhibiting NPC differentiation (Graham et al., 2003; Favaro et al., 2009). A major decrease of Sox2 was observed in the MZD E14 and E19 embryos, suggesting that Sox2 downregulation can contribute to the observed decrease in NPC proliferation. On the other hand, Sox2 downregulation causes premature neuronal differentiation (Graham et al., 2003), which was not observed in the MZD embryonic brain. Pax6 expression at E14 was also impaired in the MZD group. Pax6, a homeobox and paired domain transcription factor, promotes the expression of Tbr2, inducing the transition of NPC-like RGPs to IPCs (Sansom et al., 2009). Pax6 regulates the proliferation of cortical progenitors being expressed in RGPs at the VZ, but not in IPCs (Manuel et al., 2015). Thus, not only ERK1/2 but also Sox2 and Pax6 downregulation at a period of active progenitor proliferation can contribute to the decrease in the number of fetal brain NPCs as a consequence of maternal marginal zinc deficiency.

We next evaluated the potential disruption of the process of neuronal differentiation and specification that could be associated with an impaired NPC proliferation. Similarly to Sox2 and Pax6, Tbr2 protein levels were markedly low in E14 and E19 MZD brain CT. Tbr2 is expressed in the mouse brain as early as E10.5 in proliferative areas, where neuronal progenitors reside (Kimura et al., 1999). Tbr1 expression is located in the cerebral cortex and other brain areas populated by postmitotic neurons, decreasing the expression postnatally (Bulfone et al., 1995). Tbr2 is a marker of IPCs, being required for the progression of RGPs to IPCs, a process that is in part mediated by the downregulation of Pax6 by Trb2 (Hodge et al., 2012). Most glutamatergic neurons in the brain cortex are originated from a Tbr2 positive lineage (Vasistha et al., 2015). We observed a reduction in the number of Tbr2 positive IPCs in the SVZ and of Tbr1 positive postmitotic neurons in the cortical plate. Thus, while the decrease in NPCs in the MZD embryonic brain may explain a decreased number of neurons, the observed downregulation of Tbr2 during the period of active differentiation suggests that the specification of neurons may also be affected.

As IPCs are necessary to expand the population of cortical glutamatergic neurons, we next analyzed the population of mature NeuN positive neurons and the populations of vGlut1 and GABA positive neurons at P2 and P56. We observed not only a lower number of mature neurons in the MZD mature brain but also, in agreement with alterations in Tbr2 and Tbr1 expression, an impairment in the generation of glutamatergic neurons. Very importantly, even after postnatal zinc repletion, vGlut1 expression in the MZD P56 brain cortex remained markedly affected. The mammalian neocortex has two types of neurons, glutamatergic pyramidal cells and GABAergic non-pyramidal cells (Peters and Jones, 1984; DeFelipe and Fariñas, 1992). Glutamatergic projection neurons give rise from progenitor cells in the VZ of the pallium from where they migrate radially into the neocortex. On the other hand, GABAergic interneurons are born in the subpallium and migrate tangentially into the neocortex (Marin, 2013). They arise from the ventral telencephalon (Anderson et al., 1997), in particular the medial and caudal ganglionic eminences (Wichterle et al., 2001; Wonders and Anderson, 2006; Wonders et al., 2008) from Nkx2.1-expressing progenitors (Butt et al., 2005), in a process modulated by Sonic hedgehog (Hebert and Fishell, 2008). The described diverse origin and regulation of glutamatergic and GABAergic neurons can explain the differential effect of developmental zinc deficiency on these neuronal populations.

The current findings stress the concept that the adverse effects of limited zinc availability during early development can have irreversible consequences in the number and specification of neurons in the mature brain. These results add to previous evidence showing that severe zinc deficiency can disrupt NPC proliferation and neuronal differentiation (Swenerton et al., 1969; Dvergsten et al., 1983; Gower-Winter et al., 2013), but in a condition of marginal zinc availability which can be extrapolated to human populations. The disruption observed in neurogenesis could contribute to the described persistent effects of marginal zinc deficiency on behavior. In this regard, marginal zinc deficiency throughout gestation and lactation decreases working memory in rats after dietary repletion (Halas et al., 1986). In mice, marginal zinc deficiency through gestation leads to increased anxiety-like behavior, abnormal social behavior, and impaired motor learning even after dietary repletion (Grabrucker et al., 2014, 2016). Very relevant to the current results, a dysbalance in excitatory/inhibitory systems has been proposed as a developmental precursor of autism spectrum disorders (Mariani et al., 2015) and a possible target for restoring functional connectivity even in adulthood (Ajram et al., 2017).

In summary, this work demonstrates that gestational marginal zinc deficiency affects neurogenesis in the fetal rat brain leading to a disruption of the cortical excitatory/inhibitory balance that persists into adulthood even after dietary repletion. While earlier studies focused on severely deficient animal models, this is the first study to report disruption of fetal neurogenesis resulting from marginal zinc deficiency that affects the cellularity of the mature brain cortex. These findings stress the relevance of an adequate zinc nutrition during pregnancy to prevent irreversible effects on the offspring cortical structure and ultimately on behavior and cognition.

## Data Availability

The datasets generated for this study are available on request to the corresponding author.

## Author Contributions

PO and AA designed the research. XL, AA, SS, JN and PM performed the research. All authors participated in the analysis and discussion of data. PO, AA and JN wrote the article.

## Conflict of Interest Statement

The authors declare that the research was conducted in the absence of any commercial or financial relationships that could be construed as a potential conflict of interest.

The reviewer HB declared a shared affiliation, though no other collaboration, with several of the authors AA, PM to the handling Editor.
